# Physical Activity and Self-Determination towards Exercise among Esports Athletes

**DOI:** 10.1186/s40798-024-00700-0

**Published:** 2024-04-16

**Authors:** Mitchell Nicholson, Courtney Thompson, Dylan Poulus, Toby Pavey, Rob Robergs, Vincent Kelly, Craig McNulty

**Affiliations:** 1 School of Exercise and Nutrition Sciences, Faculty of Health, Queensland University of Technology, Brisbane, Queensland Australia; 2 School of Health, Faculty of Health, University of the Sunshine Coast, Sippy Downs, Queensland Australia; 3 School of Human Sciences, Faculty of Health, Southern Cross University, Coolangatta, Queensland Australia; 4 Manna Institute, Southern Cross University, Lismore, Australia; 5 Faculty of Health, School of Exercise and Nutrition Sciences, Queensland University of Technology (QUT), Victoria Park Road, Kelvin Grove, Queensland 4059 Australia

**Keywords:** Competitive Gaming, Movement, Physical Exertion, Behavioural Management, Physical Training

## Abstract

**Background:**

Esports is competitive video gaming, performed within teams or individually, across multiple genres. Players are required to be sedentary for extended periods and require a high-level of cognitive skills for successful competitive performance. There are conflicting findings within the physical activity research in the esports industry. The aim of this research is to explore self-reported physical activity through accelerometer-assessed physical activity, to gain a better insight into the physical activity behaviours of international e’athletes.

**Method:**

Participants (*n* = 796) across multiple popular esports games, holding any in-game rank, competing at any level, were recruited. The survey consisted of demographic details, esports experience, the International Physical Activity Questionnaire-Long Form (IPAQ-LF), and Behavioural Regulations towards Exercise Questionnaire (BREQ-3). Within a convenience sample, local intervarsity e’athletes (*n* = 18) were recruited to wear a wrist-worn accelerometer to measure physical activity for 7-days and then complete the survey. Results from the accelerometers were compared to the survey results to explore physical activity reporting within this population.

**Results:**

When comparing IPAQ-LF to accelerometer data, players significantly over-report moderate-to-vigorous physical activity and weekly MET-min^− 1^ (*p* = .018, *r* = .63 and *p* ≤ .001, *r* = .92). The BREQ-3 showed that e’athletes categorised as high physical activity displayed significantly higher levels of intrinsic motivation, when compared to players categorised as low and moderate physical activity.

**Conclusions:**

E’athletes significantly over report physical activity time when measured through the IPAQ-LF, suggesting previous surveys may overestimate physical activity and further research is needed. Given the exponential growth of the industry and the level of physical inactivity, esports may contribute to global physical inactivity levels.

**Supplementary Information:**

The online version contains supplementary material available at 10.1186/s40798-024-00700-0.

## Key Points

Despite in-game rank not influencing physical activity time or motivation, higher levels of intrinsic motivation were associated with increased physical activity among e’athletes. This suggests the importance of fostering intrinsic motivation to promote sustained engagement in physical activity within esports communities.

The study reveals concerning levels of sedentary behaviour among e’athletes, with implications for their overall health and well-being. Addressing sedentary behaviour and promoting light physical activity are crucial for mitigating associated health risks within the esports population.

The study highlights significant disparities between self-reported physical activity data and accelerometer-assessed data among e’athletes, indicating potential over-estimation of physical activity levels when self-reported.

**Supplementary Information:**

The online version contains supplementary material available at 10.1186/s40798-024-00700-0.

## Background

Esports, short for electronic sports, can be defined as “*organised competitive digital gaming, played on a spectrum of professionalism*…” [1]. The industry is growing exponentially year-on-year, with global viewership of esports enthusiasts expected to grow to 318 million by 2025, and it was estimated in 2020 the net worth is USD $24.9B [2]. Esports are now being supported by professional sporting associations [3], government or national organisations [4], and education sectors [5, 6]. This type of competition requires players to be seated for long periods, and success is often determined by perceptual-cognitive skills and fine-motor coordination [7]. This surge in global popularity may pose several health concerns regarding sedentary behaviour, and the psychological and physical well-being of those participating in esports.

The World Health Organisation (WHO) physical activity guidelines recommend that adults aged 18–64 years of age should do at least 150–300 min of moderate-intensity aerobic physical activity; or at least 75–100 min of vigorous-intensity aerobic physical activity; or a combination of moderate- and vigorous-intensity activity throughout the week. For adolescents aged 5–17 years, the WHO physical activity guidelines recommend that they should do at least an average of 60 min per day of moderate-to-vigorous intensity physical activity (MVPA; [8]). Emerging research into the physical activity behaviours of the esports industry shows conflicting findings across studies. Several studies report players exceeding the WHO physical activity guidelines [9–13], while some studies report players performing limited physical activity or not reaching guidelines at all [14–17]. The questionnaires used to quantify physical activity within esports populations have not been validated, which highlights the need for research to explore the validity of self-reported physical activity through accelerometer-assessed physical activity within esports athletes (e’athletes; [18]).

With the development of new technologies, sitting and reclining whilst engaging in specific sedentary activities (e.g., screen-based behaviours) has emerged as a major risk factor towards decreased adult and adolescent health and well-being [19, 20]. Sedentary behaviour is defined as any waking behaviour that is characterised by an energy expenditure of ≤ 1.5 metabolic equivalents of task (MET), such as sitting, reclining, or lying down [21], where a MET represents the amount of energy expended carrying out physical activity, which is 3.5 ml·O_2_·kg^− 1^·min^− 1^ at rest [22]. E’athletes and coaches believe that they need to “grind” through countless hours of gameplay in order to succeed, resulting in extended periods of sedentary time [23]. Sedentary time varies across studies with reports of up to 7.7 h a day [9, 24], while others report players sitting for up to 9 to 10 h a day [14, 17].

Self-determination theory and its sub-theory of organismic integration theory describes motivation across a continuum, differentiating between extrinsic and intrinsic motivation, and identify different types of motivation towards performing certain behaviours [25–27]. Intrinsic motivation pertains to engaging in an activity due to its inherent gratification, whereas extrinsic motivation involves undertaking an activity with the intention of achieving external rewards or outcomes detached from the satisfaction of the activity itself [28]. To gain an understanding of human motivation towards exercise participation, self-determination theory provides an understanding into the initiation and persistence of undertaking exercise [29]. Self-determination theory identifies when individuals are intrinsically motivated, which is vital for long-term exercise participation [30, 31]. Whereas extrinsic motivation, such as personally valuing specific exercise outcomes, is a crucial factor in the initial adoption process [31]. It is important to identify behavioural regulations towards exercise to promote internalised extrinsic motivations or enhanced intrinsic motivations to support perceived competence in exercise. Multiple studies have investigated e’athletes motivations towards becoming professional players [32–34], with some discussing motivation towards physical activity [12]. E’athletes are met with extrinsic motivations, such as playing for a salary or prize money, and intrinsic motivations where players compete for enjoyment, leisure, or socialisation [33]. Research has identified higher levels of motivation being exhibited during higher frequencies of playing time, displaying a greater prioritisation of esports training over physical activity or exercise [12, 35]. However, no research has investigated how motivation affects behavioural regulation towards exercise participation within esports, which may assist with health promotion within the industry.

This study has two aims: (1) to explore how in-game rank influences physical activity time and motivation towards exercise and how motivation affects physical activity time in international e’athletes; and (2) to explore how self-reported physical activity time compares to accelerometer-assessed physical activity time within a convenience sample of e’athletes. We hypothesise that higher ranked e’athletes will report greater physical activity levels and greater motivations towards exercise when compared to lower-ranked players; and that accelerometer-assessed physical activity will show e’athletes over-report weekly physical activity time.

## Methodology

### Survey Participants and Recruitment

Participant recruitment was performed via online platforms consisting of esports specific Subreddits (Reddit.com), social media (LinkedIn, Facebook, Instagram, Twitter), and Discord channels, and through emails to team support staff. The survey was designed using online cloud survey software, Qualtrics (Prove, Utah, United States), and distributed via an anonymous link online, in the English language. The survey was promoted fortnightly on these various channels for the duration of the live survey. All e’athletes over the age of 18 were eligible to participate, as e’athletes comprise all individuals who engage in esports with the goal of attaining an in-game ranking or participating in formal competitions [18]. The survey was open for four months (August 2022 to November 2022).

### Accelerometer Participants and Recruitment

A convenience sample of participants from an intervarsity esports’ academy in Queensland, Australia, were asked to wear an accelerometer (ActiGraph GT9X, Florida, USA) for 24-hours a day over seven consecutive days. Participants had to be over the age of 18 and apparently healthy to participate, with no prior medical history that would affect their normal physical activity level. Participants were also asked to complete the online survey after they had completed the 7-days of wearing the accelerometer, to recall their physical activity during the period of monitoring.

### Survey Measures

The survey consisted of four parts; demographics, esports experience, International Physical Activity Questionnaire- Long Form (IPAQ-LF), and The Behavioural Regulations in Exercise Questionnaire 3 (BREQ-3; Additional File 1; The Survey).

### Demographics

The first section of the survey collected demographic information (location, occupation status and education level). Responses for occupation status and education level were categorised according to the Australian Bureau of Statistics recommendations for reporting. Through self-reported height (cm) and weight (kg) measurements, a BMI (kg/m^2^) was calculated to report a participant descriptive.

### Esports Experience

The survey asked participants which esports title they were highest ranked in by in-game ranking. Every esports game categorises players into distinct skill tiers (or in-game ranks) based on in-game algorithms, utilising various in-game metrics such as (but not exclusive to) the number of victories and defeats [36]. This triggered a display logic within the survey to ask the participant what their specific rank was for their respective game title. Specific games titles that were listed within the survey were *League of Legends*, *Counter Strike: Global Offensive*, *Overwatch, Valorant, Apex Legends*, *Defence of the Ancients 2*, *Rainbow Six Siege*, *Starcraft 2*, *Hearthstone, Rocket League, Player Unknown Battle Grounds, Teamfight Tactics*, and a ‘other’ free text entry box for non-listed games. Some games were not listed, such as *Call of Duty* and *Fortnite*, as these games do not have clear in-game rankings, however, players can still be competing within these titles. In-game ranks across games do not align and are determined by specific in-game algorithms, which uses variable in-game statistics. Previous research has standardised players into categories based on their skill group and the player distribution across ranks for each given game, as follows: category 1 (99–100%), category 2 (90-98.9%), category 3 (80-89.9%), category 4 (70-79.9%), category 5 (60-69.9%) and category 6 (< 59.9%) [37]. These categories were determined by player distribution reports online, where reported in-game rank was transformed to a category based on the game responders competed in- defined as esports category [38–46]. Two esports (StarCraft2 and Hearthstone) did not have up-to-date player distributions of rank, resulting in exclusion from any rank analysis. Participants were asked to select which level of esport best described their current level of competition as defined by Hedlund and colleagues [47]. Participants were given the choice to answer if they played for a gaming organisation/university, and if they had any competition winnings. These questions were utilised to accurately represent the individual’s level of competition, as in-game Elo rank alone may not identify some high-level players as not all players play a competitive in-game playlist.

### International Physical Activity Questionnaire- Long Form Scoring and Data Processing

Self-reported physical activity time was assessed through the IPAQ-LF. Scoring of this questionnaire follows the IPAQ-LF guidelines for data processing and analysis to report physical activity as a continuous variable in MET-min^− 1^ a week and categorical descriptors of level of activity level [48]. The IPAQ-LF was constructed to measure health-related physical activity young and middle-aged adults (aged 15–69 years old) [22], showing good validity and reliability when implemented within a 12-country reliability test, with all versions of the questionnaire producing repeatable data (Spearman’s correlation coefficient = 0.81, for the long form) [49].

### The Behavioural Regulations in Exercise Questionnaire-3 (BREQ-3)

The level of self-determination towards exercise was measured through the BREQ-3 [50, 51]. Multidimensional scoring was performed by calculating the mean score for each set of items within each of the six dimensions, alongside calculating relative autonomy index (RAI) [31]. The BREQ-3 holds good construct validity and test-retest reliability when assessing individuals’ behavioural regulation in exercise contexts, displayed through intraclass correlation coefficients ranging from 0.70 to 0.88 across subscales [51].

### Accelerometer Measures and Processing

Accelerometer-assessed physical activity was measured through the ActiGraph Link GT9X (ActiGraph, Pensacola, FL, USA) which is a small (3.5 × 3.5 × 100 mm), and lightweight (14 g) device, worn on the non-dominant wrist. Wrist worn accelerometery was chosen over hip-worn accelerometers as wear compliance is higher and participant burden is lower for wrist worn accelerometry [52]. The accelerometer was set to 30 Hz sampling rate, with the watch screen programmed to be left blank to minimise physical activity -related feedback that may influence physical activity performance. The participants were instructed to wear the device for 7-days, including sleep time, with removal during prolonged water submersion (i.e., swimming). Data were considered valid if the watch was worn for at least 4-days, including at least 1-day of the weekend, and for at least 10 h each day [53]. Accelerometer-assessed physical activity, measured by the ActiGraph Link GT9X has been shown to be a valid and reliable measurement of free-living physical activity within children [54] and adults [55].

The device monitors the participants movement through 3 orthogonal axes that measures daily minutes within different intensities. The raw accelerometer data was downloaded from the devices through accompanying software, ActiLife v6.13.4 (ActiGraph, Pensacola, FL, USA), as a ActiGraph.gt3x file and converted to a raw time-stamp free .csv file for data processing. The data files were then processed within R statistical software ( v2023.03.1 + 446, R Core Team, Vienna, Austria) using the GGIR code package available on GitHub, the methods of this code is presented by the creators Migueles et al. [56]. The accelerometers were used to monitor sedentary time, light intensity physical activity (LPA), and moderate-to-vigorous intensity physical activity (MVPA). The following intensities are based on established cut-points in milligravity (mg): ST (0–30 mg), LPA (30 mg - <120 mg), and MVPA (> 120mg) [57]. Prior research has shown that the cut point of 120 mg for MVPA is more stringent [58–60]. The data was processed to produce both 1-minute and 10-minute bouted physical activity within each intensity. Total MET-min^− 1^ will also be calculated for both 1-minute and 10-minute bouts.

### Statistical Analysis

The survey was exported from Qualtrics as a .csv file and opened in Microsoft Excel where the data was screened for outliers and unrealistic responses. Upon assessing normality of the IPAQ-LF data through histograms, total physical activity (Total MET-min^− 1^) showed several extreme values and were excluded from the dataset (*n* = 29 excluded), following the guidelines for data processing and analysis of the IPAQ [48]. Excluded data points exceeded 16 h of activity a day, where the guidelines assume 8 h a day is spent sleeping. Data analysis was performed within Jamovi (version 2.3.18.0). Using the Shapiro-Wilks test of normality (*p* < .05), also considering skewness and Kurtosis, the physical activity data and BREQ-3 data was non-normally distributed. Therefore, the non-parametric one-way analysis of variance, Kruskal Wallis test was used to compare medians for continuous variables across subgroups, with the median and interquartile range (IQR) used for descriptive statistics. Correlations between accelerometer-assessed data and BREQ-3 was assessed through a non-parametric Spearman’s correlation. Spearman’s correlation threshold was determined as 0.90 to 1.00 as very high correlation, 0.70 to 0.90 as high correlation, 0.50 to 0.70 as moderate correlation, 0.30 to 0.50 as low correlation, and 0.00 to 0.30 as negligible correlation [61]. Significance was determined when *p ≤ .*05.

### Ethics Approval

This study was conducted according to the guidelines presented in the Declaration of Helsinki and all procedures were approved by Queensland University of Technology Human Research Ethics Committee- approval number is LR 2022-1045-10084.

## Results

### Self-Reported Physical Activity and BREQ-3 Data

#### Survey Data and Participant Characteristics

1,219 participants opened the survey. All responses were screened for legitimacy and accuracy, resulting in 423 (34.7%) responses excluded as they contained inappropriate or unreliable responses. Of the included responses (*N* = 796), 349 (43.9%) participants partially completed the survey and 447 (56.2%) participants provided full responses to the survey. The data from partial responses were reported and then excluded listwise from any analysis where full datasets were not complete. Responses came from 62 different countries across the sample (Additional File 1; Table 1: Frequency table of player country of residence for survey participants). The top five represented countries were United States (*n* = 244; 32.9%), Australia (*n* = 113; 15.2%), Germany (*n* = 53; 7.1%), Canada (*n* = 47; 6.2%), and United Kingdom (*n* = 44; 5.9%). Table 1 shows that participants predominantly competed in Overwatch (*n* = 153; 21.0%), League of Legends (*n* = 119; 16.3%), DOTA2 (*n* = 84; 11.5%), and Valorant (*n* = 60; 8.2%).

**Table 1:** Participants esports descriptive and frequency statistics and esports experience

	Total	Category 1	Category 2	Category 3	Category 4	Category 5	Category 6
**Age (Median IQR)**	23 (7)	22 (6)	22 (6)	22 (5)	25 (9)	23 (9)	23 (7)
**Gender (N** ***=*** **796)**							
Male (*n* %)	723 (90.8)	152 (26.2)	159 (27.4)	92 (15.9)	44 (7.6)	34 (5.9)	39 (6.7)
Female (*n* %)	45 (5.7)	8 (1.2)	12 (1.8)	5 (0.8)	4 (0.6)	3 (0.5)	12 (1.8)
Other (*n* %)	28 (8.8)	4 (0.7)	5 (0.9)	6 (1)	3 (0.5)	1 (0.2)	3 (0.6)
BMI (Median IQR)	24.2 (5.65)	23.6 (5.89)	23.7 (5.62)	24.5 (4.93)	25.5 (4.49)	22.9 (3.42)	23.8 (6.64)
*p-*value	0.169						
**Esports Title (*n =*** **728)**	**573 (100)**	**152 (24.5)**	**174 (26.3)**	**104 (16)**	**51 (7.9)**	**38 (5.8)**	**54 (8.3)**
Overwatch (*n* %)	153 (21.0)	29 (5.0)	39 (6.7)	43 (7.4)	20 (3.4)	14 (2.4)	7 (1.2)
League of Legends (*n* %)	119 (16.3)	28 (4.8)	36 (6.2)	15 (2.6)	16 (2.8)	6 (1.0)	16 (2.8)
DOTA 2 (*n* %)	84 (11.5)	18 (3.1)	27 (4.7)	11 (1.9)	5 (0.9)	4 (0.7)	17 (2.9)
Valorant (*n* %)	60 (8.2)	12 (1.8)	21 (3.2)	5 (0.8)	3 (0.5)	9 (1.4)	10 (1.5)
Rainbow Six Siege (*n* %)	37 (5.0)	14 (2.4)	10 (1.7)	9 (1.6)	2 (0.3)	2 (0.3)	0 (0)
Apex Legends (*n* %)	36 (4.9)	10 (1.7)	15 (2.6)	7 (1.2)	1 (0.2)	1 (0.2)	1 (0.2)
CSGO (*n* %)	35 (4.8)	9 (1.6)	12 (2.1)	9 (1.6)	3 (0.5)	2 (0.3)	0 (0)
Teamfight Tactics (*n* %)	35 (4.8)	21 (3.6)	12 (2.1)	1 (0.2)	1 (0.2)	0 (0)	0 (0)
Rocket League (*n* %)	25 (3.4)	20 (3.4)	2 (0.3)	1 (0.2)	0 (0)	0 (0)	0 (0)
PUBG (*n* %)	4 (0.5)	1 (0.2)	0 (0)	2 (0.3)	0 (0)	0 (0)	1 (0.2)
StarCraft 2 (*n* %)	55 (7.5)						
Hearthstone (*n* %)	26 (3.5)						
Other (*n* %)	59 (8.1)						
**Esports Level (*n*** **= 680)**							
Professional (*n* %)	47 (6.9)	24 (4.8)	5 (1.0)	3 (0.6)	1 (0.2)	0 (0)	0 (0)
Collegiate/ Intervarsity (*n* %)	107 (15.7)	43 (8.5)	29 (5.8)	12 (2.4)	1 (0.2)	5 (1.0)	4 (0.8)
Casual (*n* %)	469 (69.0)	66 (13.1)	101 (20.0)	70 (13.9)	39 (7.7)	23 (4.6)	37 (7.3)
High School (*n* %)	13 (1.9)	1 (0.2)	4 (0.8)	0 (0.0)	1 (0.2)	0 (0.0)	1 (0.2)
Youth (*n* %)	44 (6.5)	8 (1.6)	12 (2.4)	8 (1.6)	1 (0.2)	2 (0.4)	3 (0.6)

*Note* Category 1 = 99–100%, Category 2 = 90-98.9%, Category 3 = 80-89.9%, Category 4 = 70-79.9%, Category 5 = 60-69.9%, Category 6 = < 59.9%. StarCraft 2 and Hearthstone do not have player rank distributions available- excluded from rank analysis

#### Physical Activity Scores from the IPAQ-LF

Table 2 demonstrates that e’athletes reported a median (IQR) 2916 (4400) MET-min^− 1^ of physical activity. These reports resulted in 61.4% of the population being classified as performing a ‘high’ amount of physical activity a week, 27.8% being classified as moderate physical activity and 10.8% being classified as low physical activity. A Kruskal-Wallis test demonstrated no significant differences across esports categories for any measurement of physical activity- total MET-min^− 1^*χ*^2^_5_ = 5.1, *p* = .40; MVPA *χ*^2^_5_ = 5.7, *p* = .33; sedentary time *χ*^2^_5_ = 2.5, *p =* .78; LPA *χ*^2^_5_ = 4.5, *p = .*48; activity level *χ*^2^_5_ = 3.2, *p =* .67.

**Table 2:** IPAQ-LF scores and descriptive statistics

	Total	Category 1	Category 2	Category 3	Category 4	Category 5	Category 6	
**Physical Activity (*n*** = **518)**								
Physical Activity MET-min^− 1^/wk (Median IQR)	2916 (4400)	2981.3 (4123)	3049.5 (4361)	2613 (3863)	3280 (5676)	2478 (3288)	2373 (4551)	
*p-value*	0.41							
MVPA mins/week (Median IQR)	120 (161)	90 (126)	120 (146)	110 (160)	120 (146)	118 (180)	135 (191)	
*p-value*	0.33							
LPA mins/week (Median IQR)	60 (90)	50 (65)	60 (100)	57.5 (86.3)	60 (81.3)	84.5 (87.5)	60 (78.8)	
*p-value*	0.48							
**Level of Activity (*n =*** **518*)***								
High Activity (*n* %)	318 (61.4)	81 (14.3)	93 (16.4)	48 (8.5)	32 (5.6)	12 (2.1)	19 (3.4)	
Moderate Activity (*n* %)	144 (27.8)	36 (6.3)	37 (6.5)	20 (3.5)	11 (1.9)	8 (1.4)	11 (1.9)	
Low Activity (*n* %)	56 (10.8)	39 (6.9)	41 (7.2)	33 (5.8)	7 (1.2)	18 (3.2)	21 (3.7)	
**Sedentary Time (*n*** **= 470)**								
Sedentary Time mins/days- Median (IQR)	600 (418)	629 (403)	609 (454)	617 (394)	600 (480)	531 (326)	549 (291)	
*p-value*	0.78							

*Note* IQR, Interquartile Range; n %, number and percentage of total participants; MVPA, Moderate-to-Vigorous Physical Activity; LPA, Light Physical Activity

#### BREQ-3 Results within International Esports Participants

Table 3 shows the group’s median (IQR) RAI was 6 (6.50), with the lowest scores observed within the dimension of *amotivation* (Median [IQR] = 1.00 [0.5]), and highest scores were observed within *identified regulation* (Median [IQR] = 4.00 [1.50]). The Kruskal-Wallis test revealed no statistical difference between esports level, and all dimensions measured through the BREQ-3. Participants categorised as high physical activity demonstrated significantly higher RAI (RAI = 7.25 [5.63]; *χ*^2^_2_ = 41.43, *p = < .*001) when compared to low (RAI = 3.13 [4.81]) and moderate physical activity (RAI = 3.50 [7.50]) participants. Scores of *amotivation* were significantly lower (*p* *= < .*001) within high physical activity participants when compared to low physical activity participants. High physical activity participants demonstrated significantly higher scores (*p = < .*001) within *identified regulation*, *integrated regulation*, and *intrinsic regulation*, when compared to both moderate and low physical activity groups.

**Table 3:** BREQ-3 scores: a comparison across esports level and physical activity level

	RAI Median (IQR)	Amotivation Median (IQR)	External Regulation Median (IQR)	Introjected Regulation Median (IQR)	Identified Regulation Median (IQR)	Integrated Regulation Median (IQR)	Intrinsic Regulation Median (IQR)
All E’athletes (*n* = 447)	6.00 (6.50)	1.00 (0.5)	1.50 (1.25)	3.00 (2.00)	4.00 (1.50)	2.75 (2.00)	3.50 (2.00)
**Esports Category (*n*** = **447)**							
Category 1 (*n* = 96)	5.63 (6.63)	1.00 (0.75)	1.50 (1.00)	3.00 (2.25)	3.50 (1.00)	2.50 (1.75)	3.25 (1.81)
Category 2 (*n* = 100)	5.88 (6.00)	1.00 (0.50)	1.50 (1.25)	3.00 (2.25)	3.75 (1.50)	2.75 (1.75)	3.25 (1.81)
Category 3 (*n* = 55)	5.25 (7.38)	1.00 (0.50)	1.25 (1.13)	3.25 (1.63)	4.00 (1.63)	2.50 (1.63)	3.00 (2.00)
Category 4 (*n* = 25)	7.25 (7.25)	1.00 (0.50)	1.50 (1.25)	3.00 (1.75)	4.00 (1.25)	2.75 (1.50)	3.50 (1.75)
Category 5 (*n* = 19)	5.75 (9.63)	1.00 (0.25)	1.75 (1.63)	3.50 (1.63)	3.75 (1.88)	2.50 (2.75)	4.00 (2.88)
Category 6 (*n* = 27)	4.25 (7.88)	1.00 (0.50)	1.75 (1.50)	3.00 (1.50)	3.75 (1.50)	2.50 (1.63)	3.50 (3.13)
*p-value*	0.89	0.92	0.53	0.69	0.52	0.75	0.68
**Physical Activity Category (*n*** = **364)**							
High (*n* = 255)	7.25 (5.63) ^*, #^	1.00 (0.25) ^*^	1.25 (1.00)	3.00 (1.75)	4.00 (1.25) ^*, #^	3.00 (1.75) ^*, #^	3.75 (1.50) ^*, #^
Moderate (*n* = 109)	3.50 (7.50) ^*^	1.25 (1.00)	1.50 (1.25)	3.00 (2.00)	3.25 (1.50) ^*^	2.25 (1.50) ^*^	2.50 (2.25) ^*^
Low (*n* = 30)	3.13 (4.81) ^#^	1.25 (0.75) ^*^	1.25 (1.00)	3.13 (2.19)	3.25 (0.75) ^#^	2.00 (1.44) ^#^	2.63 (1.88) ^#^
*p-value*	< 0.001	< 0.001	0.08	0.09	< 0.001	< 0.001	< 0.001

*Note* IQR = Interquartile Range, “^*^ and ^#^” denotes groups that are significantly different in columns (*p* = < 0.05). RAI = Relative Autonomy Index, Category 1 = 99 – 100%, Category 2 = 90 - 98.9%, Category 3 = 80 - 89.9%, Category 4 = 70 - 79.9%, Category 5 = 60 - 69.9%, Category 6 = < 59.9%

### Accelerometer-Assessed Physical Activity Data

#### Accelerometer-Assessed Participant Characteristics

The survey results of the participants who agreed to wear the accelerometers appear in Table 4. One was excluded due to a corrupt accelerometer file (*n* = 18).

**Table 4:** Accelerometer-assessed participant characteristics

	Total (*n* = 18)	Category 1	Category 2	Category 3	Category 4	Category 6
**Age (Median IQR)**	20 (3.50)	20 (2.0)	19 (1.0)	25 (0.0)	20 (0.0)	23.5 (5.50)
**Gender**						
Male (n %)	13 (72.2)	7 (38.9)	5 (27.8)	-	1 (5.6)	-
Female (n %)	5 (27.8)	-	-	1 (5.6)	-	4 (22.2)
BMI (Median IQR)	23.4 (5.76)	26.1 (9.40)	20.5 (1.39)	25.2 (0.0)	24.6 (0.0)	23.9 (5.21)
*p-value*	0.48					
**Esports Title**						
League of Legends (n %)	13 (66.7)	5 (27.8)	2 (16.7)	1 (5.6)	-	3 (16.7)
Overwatch (n %)	3 (16.7)	1 (5.6)	-	-	-	1 (5.6)
Valorant (n %)	2 (11.1)	1 (5.6)	1 (5.6)	-	-	-
Rocket League (n %)	1 (5.6)	-	1 (5.6)	-	-	-
**Esports Level**						
Professional	1 (5.6)	1 (5.6)	-	-	-	-
Collegiate/ Intervarsity	14 (72.2)	5 (27.8)	4 (22.2)	1 (5.6)	-	3 (16.7)
Casual	4 (22.2)	1 (5.6)	1 (5.6)	-	1 (5.6)	1 (5.6)
**BREQ-3**						
RAI	5.25 (6.13)	5.50 (7.25)	4.00 (3.25)	-2.25 (0.00)	5.50 (0.00)	6.75 (4.44)
Intrinsic Regulation	3.50 (1.31)	3.50 (1.63)	3.75 (1.00)	2.75 (0.00)	3.00 (0.00)	3.25 (1.13)
Integrated Regulation	2.50 (2.38)	2.50 (1.88)	3.25 (2.25)	2.00 (0.00)	2.00 (0.00)	3.50 (1.50)
Identified Regulation	3.50 (1.44)	3.50 (1.00)	3.50 (2.00)	2.25 (0.00)	2.50 (0.00)	3.88 (0.44)
Introjected Regulation	2.75 (1.44)	2.75 (1.00)	2.75 (2.25)	2.50 (0.00)	1.00 (0.00)	2.63 (1.56)
External Regulation	1.75 (2.00)	1.50 (1.38)	2.00 (1.25)	3.75 (0.00)	2.00 (0.00)	1.00 (0.81)
Amotivation	1.38 (0.75)	1.50 (0.62)	1.75 (1.00)	1.75 (0.00)	1.00 (0.00)	1.00 (0.06)

*Note* No participants had an in-game rank categorised within category 5. ‘-’ no participants within that group. RAI = Relative Autonomy Index; BREQ-3 = Behavioural Regulations in Exercise Questionnaire-3

#### Accelerometer-Assessed vs. IPAQ-LF Physical Activity

When comparing self-reported data with the 10-minute bout accelerometer-assessed physical activity data (AT10; Table 5), the Wilcoxon paired t-test revealed significant differences between for MVPA (*p* = .01) and MET-min^− 1^ per week (*p* < .001). Effect sizes were moderate, ranging from *r*=-.12 for LPA to *r* = .92 for MET-min^− 1^ per week. Resulting in significant over-reporting of median MVPA time by 26.3 min and median weekly MET-min^− 1^ by 3128 MET-min^− 1^ per week.

When comparing self-reported physical activity data to 1-minute bout accelerometer-assessed physical activity data (AT1; Table 5), the Wilcoxon paired t-test showed highly significant differences for LPA (*p* < .001) and MET minutes per week (*p* = .02). Resulting in an over-reporting of 15 min per day of MVPA and 1647 MET-min^− 1^ per week, and an under reporting of LPA by 49.7 min per day when compared to AT1 data. Effect sizes varied, with a large effect size of *r *= -.95 for LPA and a moderate effect size of *r* = .48 for MVPA.

**Table 5:** Descriptive statistics and statistical differences of Accelerometer 10-min (AT10) and 1-min bout (AT1) data to IPAQ-LF for LPA, MVPA, MET-min^-1^, and Sedentary Time

Components	IPAQ-LF Median (IQR)	AT10 Median (IQR)	Inference Statistics			AT1 Median (IQR)	Inference Statistics		
			p-value	Effect Size (r)	W		p-value	Effect Size (r)	W
LPA (mins/day)	12.9 (25.4) ^#^	14.0 (23.4)	0.67	-0.12	67.0	62.6 (70.5) ^#^	< 0.001^#^	-0.95	4.0
MVPA (mins/day)	33.2 (57.9) ^*^	6.90 (10.9) ^*^	0.02^*^	0.63	139.0	18.2 (11.9)	0.07	0.48	127.0
MET-min^− 1^ /wk	3879 (4792) ^*, #^	751 (686) ^*^	< 0.001^*^	0.92	164.0	2232 (1246) ^#^	0.02^#^	0.62	139.0
Sedentary Time (mins/ day)	617 (193)	522 (322)	0.19	0.36	116.0	573 (200)	0.93	0.03	88.0

*Note* *, #: denote values that are significantly different from each other. AT10 = Accelerometer 10-min bout data; AT1 = Accelerometer 1-min bout Data; MVPA = Moderate-to-Vigorous Physical Activity; LPA = Light Physical Activity; IQR = Interquartile Range

#### Accelerometer-Assessed Physical Activity Data vs. BREQ-3

Table 6 shows a significantly high positive correlation between MET-min^− 1^ and LPA (r_s _= 0.88). It also showed a significantly high negative correlation for sedentary time between MET-min^− 1^ (r_s _= -0.72) and moderate negative correlation for LPA (r_s _= -0.66). In terms of motivation, there was a significant moderate positive correlation between MET-min^− 1^ and RAI (r_s _= 0.52), which was supported by a significant low positive correlation between integrated regulation and MET-min^− 1^ (r_s _= 0.48). Amotivation had a significant moderate negative correlation with LPA (r_s _= -0.53). Intrinsic regulation had a significant moderate positive correlation with MVPA (r_s _= 0.61).

**Table 6:** Spearman’s Correlation Matrix investigating the relationship between AT1 data and BREQ-3 Dimensions

	MET-min^− 1^	LPA	MVPA	ST	RAI	Intrinsic Regulation	Integrated Regulation	Identified Regulation	Introjected Regulation	External Regulation	Amotivation
MET-min^− 1^	—										
LPA	0.88***	—									
MVPA	0.38	0.07	—								
ST	-0.72**	-0.66**	-0.43	—							
RAI	0.52**	0.46	0.17	-0.10*	—						
Intrinsic Regulation	0.42	0.20	0.61**	-0.16**	0.58*	—					
Integrated Regulation	0.48*	0.37	0.32	-0.17**	0.49*	0.69**	—				
Identified Regulation	0.23	0.00	0.18	-0.12*	0.49*	0.57*	0.75***	—			
Introjected Regulation	0.31	0.16	0.21	-0.15**	0.25	0.49*	0.62**	0.78***	—		
External Regulation	-0.37	-0.36	0.08	-0.05	-0.79***	-0.22	0.02	-0.16	0.04	—	
Amotivation	-0.42	-0.53*	0.04	0.08	-0.56*	-0.04	-0.15	-0.09	-0.13	0.41	—

*Note*  * *p* < .05, ** *p* < .01, *** *p* < .001. LPA = Light physical activity; MVPA = Moderate-to-Vigorous Physical Activity; ST = Sedentary Time; RAI = Relative Autonomy Index

## Discussion

The current study aimed to explore how in-game rank influences physical activity time and motivation towards exercise, and how motivation affects physical activity time in international e’athletes. The results reject the hypothesis and in-game rank does not influence physical activity time or motivation towards exercise. However, e’athletes who reported higher levels of intrinsic motivation performed more physical activity. The study also aimed to explore how self-reported physical activity time compares to accelerometer-assessed physical activity time within a convenience sample of e’athletes. The results accept our hypothesis as accelerometer-assessed physical activity identified that e’athletes are significantly over-reporting physical activity time through the IPAQ-LF.

### Comparison between IPAQ-LF and Accelerometer-Assessed Data

In terms of in-game player rank, the results from this survey showed that in-game rank did not affect level of physical activity level or sedentary time. The median sedentary time from our findings are consistent with other surveys [9, 14, 17]. This is the first study to use accelerometer-assessed physical activity within an esports population, whilst simultaneously comparing the results to self-reported physical activity data. When comparing self-reported physical activity results to accelerometer-assessed data, findings show e’athletes significantly over report MVPA and weekly MET-min^− 1^, supported by moderate to large effects sizes. Our findings suggest that caution may be needed when interpreting previous self-reported findings. Over-reporting is a common issue with self-reported physical activity, as participants follow social desirability biases in recalling information [62, 63]. The IPAQ-LF is also known to over-report physical activity, which underestimates the level of physical inactivity [64–66]. The results from the 1-minute bout accelerometer-assessed physical activity also add that players are sedentary for a median 573 min per day. Increases in screen time, such as an average of 570 min or more a day, have been linked to a significantly higher risk of adverse health outcomes, including mortality [67]. Our findings are similar to that of another study within university students, showing that students had an average sedentary time of 600 ± 72 min per day [68]. However, this sample of university students also averaged 66 ± 30 min per day of MVPA and 201 ± 42 min per day of LPA. This suggests that intervarsity e’athletes are performing significantly less physical activity a day when compared to university students of similar demographic characteristics. This is supported by DiFrancisco-Donoghue et al. [15] who highlighted that collegiate level e’athletes perform significantly less physical activity per day when compared to age-matched controls.

Failing to meet the international physical activity recommendations and participating in excessive levels of sedentary time is concerning as these are major modifiable risk factors that contribute to the development of multiple non-communicable diseases [69–71]. The 1-minute bout accelerometer-assessed physical activity data correlations showed different findings, highlighting that sedentary time had significant high and moderate negative correlation between MET-min^− 1^ and LPA. This implies that as e’athletes become more sedentary, they are becoming more physically inactive, predominantly through a decrease in LPA, and they are not performing enough physical activity to mitigate extensive sedentary times. There is now extensive literature that demonstrates higher levels of physical activity at any intensity, with decreases in sedentary time, are associated with lower risks of premature mortality [72]. Additionally, Ekelund et al. [67] has shown the importance of participating in leisure time physical activity by demonstrating that high levels of moderate intensity exercise (60–75 min per day) seem to eliminate the risk of death associated with high sitting time.

The 1-minute bout accelerometer-assessed physical activity data is substantially higher than previous esports surveys reporting self-reported sedentary time [9, 10, 17, 24], with weekly MET-min^− 1^ being lower than what has been reported through previous IPAQ’s in esports populations [10, 12, 73]. These findings raise concerns that previous international esports surveys using any version of the IPAQ may be under reporting the level of physical inactivity within esports [10, 12, 24, 73]. Concerns of under reporting of physical inactivity through the IPAQ-LF have been raised within global surveillance studies [64]. For example, the IPAQ showed 90.4% of a Canadian adult sample reached physical activity guidelines, whereas, ATs showed only 28.7% of that sample met the guidelines [74]. In contrast, a study comparing IPAQ short-form physical activity to accelerometer-assessed physical activity within college students showed they over reported physical activity time by 46.24 min per day higher than what was measured by an accelerometer [75]. Within undergraduate university students, the IPAQ-LF showed to over-report MVPA by 25.6 min per day, under-report sedentary time by 134 min per day and overclassified 25% of sample as meeting physical activity guidelines [76]. This is similar to the findings of the present study, where e’athletes over-reported MVPA time on the IPAQ when compared to 10-minute and 1-minute bout accelerometer-assessed physical activity data respectively.

Our findings suggest that the IPAQ significantly over-reports physical activity time in e’athletes. Previous health survey’s using the IPAQ within esports have shown similar physical activity levels suggesting that the majority of e’athletes are exceeding the international PA guidelines [10, 12, 24, 73]. When using a different questionnaire, 66.9% of a German esports’ population reported they perform more than 2.5 h a week of MVPA, although the validity of this questionnaire is also unknown. Conversely, Trotter et al. [16] displayed that 80.3% of e’athletes are not meeting the WHO physical activity guidelines. Disparities amongst survey results are a result of using different questionnaires to quantify physical activity, as all but two of the survey’s that show e’athletes reaching the international physical activity guidelines used either the IPAQ long or short form. The IPAQ regularly over-reports physical activity, leading to an underestimation of the prevalence of insufficient activity within populations [65, 66, 77, 78]. However, future research is needed to explore under reporting in esports to better understand physical activity levels.

### Physical Activity and Dimensions of Motivation

Our results show no significant differences between esports category or any dimension of motivation towards exercise. Results from the BREQ-3 highlighted that this sample of international e’athletes reported having an RAI of 6.00 (6.50) towards exercise, which was made up of predominantly *intrinsic* regulatory dimensions. Players who have higher levels of internalised motivations, perform higher levels of exercise, when compared to players with externalised motivations or *amotivation*. Also, individuals who were highly physically active reported significantly higher scores (*p ≤* .001) within *identified regulation*, *integrated regulation*, and *intrinsic regulation*, when compared to moderate and low physical activity groups. This is consistent within university students demonstrating that weekly MET.mins have a significant positive correlation with *intrinsic motivation*, *integrate regulation* and *identified regulation*, but a significant negative correlation with *amotivation* [79]. These findings were also supported within health care university students, where scores for *identified* and *intrinsic* regulations incrementally increased with increasing levels of physical activity [80]. This aligns with internalised extrinsic regulations, where an individual values a certain outcome of exercise, which is important in the initial adoption of the behaviour [31]. Whereas the predominance of *intrinsic motivation* (i.e., valuing the experience of exercise), is important in the long-term adoption of exercise participation [31]. Interventions targeting self-determination theory, aiming to enhance autonomy and intrinsic motivation towards exercise, have been successful in multiple population [31, 81–83].

A major finding from this study is that intrinsic motivations towards exercise is a major factor towards physical activity performance and programs developing these motivations could be beneficial within esports. In line with self-determination theory, every individual possesses an inherent requirement to experience a sense of efficacy (competence), to establish meaningful connections with others (relatedness), and to have autonomy and personal endorsement in the activities they engage in, which supports intrinsic motivation [26]. Recent research has developed a classification system of motivational behaviours that support these three psychological needs of autonomy, competence, and relatedness within teaching [84]. This framework could be used for future self-determination theory-based interventions or research that aims to enhance intrinsic motivation towards exercise within esports. Also, future research needs to identify the potential facilitators and barriers towards physical activity performance as the international sample of e’athletes reported predominantly internalised regulatory dimensions.

### Limitations

A limitation of the study is that the sample did not evenly represent each category of esports experience, each game title, each region, and females were underrepresented across both self-reported physical activity and accelerometer-assessed physical activity. Future research should make every effort to encourage female e’athlete participation. It is a common issue within esports performance and health literature that female e’athletes are underrepresented [85], with 93.7% of participants being males [86]. Other limitations include not assessing barriers or limitations to physical activity performance, which could be a major limiting factor for some individuals wishing to participate in more physical activity. It is recommended that future research identifies specific facilitators and barriers (which can be individual, social, and environmental [87]) across levels of esport to enhance physical activity participation. This is especially relevant to female populations, who are less physically active than males [64] and report different facilitators and barriers towards physical activity [88]. Additionally, the inclusion and exclusion criteria of the survey did not assess health status of participants, which could have resulted in the inclusion of participants with underlying conditions preventing them from participating in physical activity. A strength of this study is the that it is the first to use accelerometers to explore self-report measures of physical activity within esports. Other strengths include a large sample size, with participants providing responses to numerous pieces of information relating to physical activity. This study provides valuable insight into the physical activity behaviours and behavioural regulation towards physical activity participation which can assist the development of interventions within practical and research settings.

### Practical Implications

The findings from this study presents multiple practical implications for promoting physical activity within the esports industry. There is clear need to utilise accurate physical activity assessment methods, such as accelerometers, to obtain valid and reliable data on physical activity levels among e’athletes. Consistently employing valid and reliable methods will enable ongoing monitoring of physical activity rates within e’athletes over time. Future interventions may consider focusing on enhancing intrinsic motivation towards exercise, as our findings highlight that individuals with higher levels of intrinsic motivation demonstrate greater levels of physical activity. Additionally, it is recommended that the sedentary behaviour among e’athletes is addressed, as high levels of sedentary time were observed in this study. Practitioners within the industry are encouraged to educate players and stakeholders on the importance of physical activity and provide strategies to incorporate regular physical activity and exercise in e’athlete routines. Future research investigating the role of physical activity and exercise on supporting esports performance to help promote exercise in esports would be beneficial. Collaboration with health professionals to develop comprehensive health promotion strategies tailored to the unique needs of e’athletes are also encouraged. Overall, implementing these practical implications will promote physical activity with the goal of improving overall health and wellbeing outcomes for e’athletes.

## Conclusion

In conclusion, the results of this study highlighted that e’athletes over report weekly physical activity time when measured through the IPAQ-LF, with lower physical activity estimates when assessed through accelerometry. Esports rank did not affect any measure of physical activity or dimension of motivation towards exercise, which is conflicting to previous results. These findings are concerning given the risk factors associated to physical inactivity and sedentary behaviour throughout aging. Future research needs to use valid and reliable measurements of physical activity to allow for direct comparison of results and monitoring of physical activity levels over time. A major finding from this study was that e’athletes who exhibited more internalised motivations towards exercise, performed more weekly physical activity, when compared to players who had more externalised motivations or amotivation towards exercise. This highlights that interventions based on self-determination theory may be beneficial in enhancing intrinsic motivation within e’athletes to promote habitual physical activity and exercise behaviours. The identification of facilitators and barriers towards physical activity and exercise, will likely also further inform health promotion within the industry. Considering the rapid expansion of the esports industry and its link to increased sedentary behaviour, acknowledging the potential impact on physical activity levels is crucial.

### Electronic Supplementary Material

Below is the link to the electronic supplementary material.

Supplementary Material 1

## Data Availability

The datasets used and/or analysed during the current study are available from the corresponding author on reasonable request.

## Abbreviations

MVPA: Moderate-to-Vigorous Physical Activity
LPA: Light Physical Activity
BREQ-3: Behavioural Regulation in Exercise Questionnaire- 3
RAI: Relative Autonomy Index
IPAQ-LF: International Physical Activity Questionnaire- Long Form
AT1: Accelerometer data in 1-minute bouts
AT10: Accelerometer data in 10-minute bouts
MET: Metabolic Equivalent of Task
BMI: Body Mass Index

## References

[1] Formosa J, O’Donnell N, Horton EM, Türkay S, Mandryk RL, Hawks M et al. Definitions of Esports: A Systematic Review and Thematic Analysis. Proceedings of the ACM on Human-Computer Interaction. 2022;6(CHI PLAY):1–45. 10.1145/3549490.

[2] Ahn J, Collis W, Jenny S. The one billion dollar myth: Methods for sizing the massively undervalued esports revenue landscape. International Journal of Esports. 2020;1(1). Available from: https://www.ijesports.org/article/15/html.

[3] Goswami S. From Reality To Virtual: Why Traditional Sports Are Getting Into Esports Forbes: Forbes; 2022 [02/11/2022]. Available from: https://www.forbes.com/sites/forbestechcouncil/2022/05/17/from-reality-to-virtual-why-traditional-sports-are-getting-into-esports/?sh=23f906974736.

[4] Chung T, Sum S, Chan M, Lai E, Cheng N. Will esports result in a higher prevalence of problematic gaming? A review of the global situation. Journal of Behavioral Addictions. 2019;8(3):384 – 94. Available from: https://akjournals.com/view/journals/2006/8/3/article-p384.xml; 10.1556/2006.8.2019.46. 10.1556/2006.8.2019.46 PMC7044624 31553236

[5] Hennick C. eSports Programs Start to Pop up in K–12 Schools 2019 [Available from: https://edtechmagazine.com/k12/article/2019/01/esports-programs-start-pop-k-12-schools.

[6] Trotter MG, Coulter TJ, Davis PA, Poulus DR, Polman R (2021). Examining the Impact of School Esports Program Participation on Student Health and Psychological Development. Front Psychol.

[7] Pedraza-Ramirez I, Musculus L, Raab M, Laborde S (2020). Setting the scientific stage for esports psychology: a systematic review. Int Rev Sport Exerc Psychol.

[8] Bull FC, Al-Ansari SS, Biddle S, Borodulin K, Buman MP, Cardon G (2020). World Health Organization 2020 guidelines on physical activity and sedentary behaviour. Br J Sports Med.

[9] Rudolf K, Bickmann P, Froböse I, Tholl C, Wechsler K, Grieben C. Demographics and Health Behavior of Video Game and eSports players in Germany: the eSports Study 2019. Int J Environ Res Public Health [Internet]. 2020; 17(6). 10.3390/ijerph17061870 PMC7142975 32183070

[10] Pereira AM, Verhagen E, Figueiredo P, Seabra A, Martins A, Brito J. Physical Activity Levels of Adult Virtual Football Players. Frontiers in Psychology. 2021;12. Available from: https://www.frontiersin.org/articles/10.3389/fpsyg.2021.596434. 10.3389/fpsyg.2021.596434. 10.3389/fpsyg.2021.596434 PMC8044777 33868076

[11] Kari T, Siutila M, Karhulahti V-M. An extended study on training and physical exercise in esports. Exploring the cognitive, social, cultural, and psychological aspects of gaming and simulations. IGI Global; 2019. pp. 270–92.

[12] Giakoni-Ramírez F, Merellano-Navarro E, Duclos-Bastías D (2022). Professional esports players: motivation and physical activity levels. Int J Environ Res Public Health.

[13] Lepp A, Dowdell B, Yim B, Barkley JE, Esports, Gamers. Recreational gamers, and the active couch potato lifestyle. Am J Lifestyle Med. 2023;15598276231184159. 10.1177/15598276231184159.

[14] DiFrancisco-Donoghue J, Balentine J, Schmidt G, Zwibel H. Managing the health of the eSport athlete: an integrated health management model. BMJ Open Sport &amp; Exercise Medicine. 2019;5(1):e000467. Available from: http://bmjopensem.bmj.com/content/5/1/e000467.abstract; 10.1136/bmjsem-2018-000467. 10.1136/bmjsem-2018-000467 PMC6350739 30792883

[15] DiFrancisco-Donoghue J, Werner WG, Douris PC, Zwibel H. Esports players, got muscle? Competitive video game players’ physical activity, body fat, bone mineral content, and muscle mass in comparison to matched controls. Journal of Sport and Health Science. 2020. Available from: https://www.sciencedirect.com/science/article/pii/S2095254620300934; 10.1016/j.jshs.2020.07.006. 10.1016/j.jshs.2020.07.006 PMC9729923 32711155

[16] Trotter MG, Coulter TJ, Davis PA, Poulus DR, Polman R (2020). The association between esports participation, health and physical activity behaviour. Int J Environ Res Public Health.

[17] Bayrakdar A, Yıldız Y, Bayraktar I (2020). Do e-athletes move? A study on physical activity level and body composition in elite e-sports. Phys Educ Students.

[18] Bubna K, Trotter MG, Polman R, Poulus DR. Terminology matters: defining the esports athlete. Frontiers in Sports and Active Living. 2023;5. Available from: https://www.frontiersin.org/articles/10.3389/fspor.2023.1232028; 10.3389/fspor.2023.1232028. 10.3389/fspor.2023.1232028 PMC10485553 37691641

[19] Saunders TJ, McIsaac T, Douillette K, Gaulton N, Hunter S, Rhodes RE (2020). Sedentary behaviour and health in adults: an overview of systematic reviews. Appl Physiol Nutr Metab.

[20] Kuzik N, da Costa BG, Hwang Y, Verswijveren SJ, Rollo S, Tremblay MS (2022). School-related sedentary behaviours and indicators of health and well-being among children and youth: a systematic review. Int J Behav Nutr Phys Activity.

[21] Tremblay MS, Aubert S, Barnes JD, Saunders TJ, Carson V, Latimer-Cheung AE (2017). Sedentary behavior research network (SBRN)–terminology consensus project process and outcome. Int J Behav Nutr Phys Activity.

[22] Hagströmer M, Oja P, Sjöström M. The International Physical Activity Questionnaire (IPAQ): a study of concurrent and construct validity. Public Health Nutrition. 2006;9(6):755 – 62. Available from: https://www.cambridge.org/core/article/international-physical-activity-questionnaire-ipaq-a-study-of-concurrent-and-construct-validity/A78914A4CFE41987A40C122FDF8BE229; 10.1079/PHN2005898. 10.1079/phn2005898 16925881

[23] Watson M, Smith D, Fenton J, Pedraza-Ramirez I, Laborde S, Cronin C. Introducing esports coaching to sport coaching (not as sport coaching). Sports Coaching Rev. 2022:1–20. 10.1080/21640629.2022.2123960.

[24] Roncone J, Kornspan AS, Hayden EW, Fay M. The relationship of physical activity and mental toughness in collegiate esports varsity student-athletes. The Ohio Association for Health• Physical Education• Recreation• Dance. 2020:2004. Available from: https://www.researchgate.net/publication/344809575_The_relationship_of_physical_activity_and_mental_toughness_in_collegiate_esports_varsity_student-athletes.

[25] Ryan RM, Deci EL (2000). Self-determination theory and the facilitation of intrinsic motivation, social development, and well-being. Am Psychol.

[26] Ryan RM, Patrick H. Self-determination theory and physical. Hellenic journal of psychology. 2009;6(2):107 – 24. Available from: https://psycnet.apa.org/record/2009-12422-002.

[27] Ryan RM, Deci EL. Overview of self-determination theory: An organismic dialectical perspective. Handbook of self-determination research. 2002;2:3–33. Available from: https://psycnet.apa.org/record/2002-01702-001.

[28] Deci EL, Ryan RM. Intrinsic motivation and self-determination in human behavior. Springer US; 2013.

[29] Wilson PM, Mack DE, Grattan KP (2008). Understanding motivation for exercise: a self-determination theory perspective. Can Psychol / Psychologie Canadienne.

[30] Weman-Josefsson K, Lindwall M, Ivarsson A (2015). Need satisfaction, motivational regulations and exercise: moderation and mediation effects. Int J Behav Nutr Phys Activity.

[31] Teixeira PJ, Carraça EV, Markland D, Silva MN, Ryan RM (2012). Exercise, physical activity, and self-determination theory: a systematic review. Int J Behav Nutr Phys Activity.

[32] Seo Y (2016). Professionalized consumption and identity transformations in the field of eSports. J Bus Res.

[33] Kim SH, Thomas MK (2015). A stage theory model of professional video game players in South Korea: the socio-cultural dimensions of the development of expertise. Asian J Inform Technol.

[34] Bányai F, Griffiths MD, Király O, Demetrovics Z (2019). The psychology of esports: a systematic literature review. J Gambl Stud.

[35] Lafrenière M-AK, Verner-Filion J, Vallerand RJ (2012). Development and validation of the Gaming Motivation Scale (GAMS). Pers Indiv Differ.

[36] Makarov I, Savostyanov D, Litvyakov B, Ignatov DI, editors. Predicting winning team and probabilistic ratings in Dota 2 and Counter-strike: global Offensive Video games. Analysis of images, Social Networks and texts; 2018 2018//; Cham: Springer International Publishing.

[37] Poulus D, Coulter TJ, Trotter MG, Polman R. Stress and Coping in Esports and the Influence of Mental Toughness. Frontiers in Psychology. 2020;11. Available from: https://www.frontiersin.org/articles/10.3389/fpsyg.2020.00628; 10.3389/fpsyg.2020.00628. 10.3389/fpsyg.2020.00628 PMC7191198 32390900

[38] Milella V. DOTA Seasonal Rank Distribution and Medals: Esports Tales; 2022 [30/05/2023]. Available from: https://www.esportstales.com/dota-2/seasonal-rank-distribution-and-mmr-medals.

[39] Milella V. League of Legends Rank Distribution in Solo Queue Esports Tales: Esports Tales; 2023 [30/05/2023]. Available from: https://www.esportstales.com/league-of-legends/rank-distribution-percentage-of-players-by-tier.

[40] Milella V. Overwatch Competitive Rank Distribution: PC and Console- Updated Monthly Esports Tales: Esports Tales; 2023 [30/05/2023]. Available from: https://www.esportstales.com/overwatch/competitive-rank-distribution-pc-and-console.

[41] Milella V. Valorant Rank Distribution and Players Percentage- May 2023 Esports Tales: Esports Tales; 2023 [30/05/2023]. Available from: https://www.esportstales.com/valorant/rank-distribution-and-percentage-of-players-by-tier.

[42] Milella V. Apex Legends Distribution and Percentage of Players by Tier- May 2023 Season 17 Esports Tales: Esports Tales; 2023 [30/05/2023]. Available from: https://www.esportstales.com/apex-legends/rank-distribution-and-percentage-of-players-by-tier.

[43] Milella V, R6S Seasonal Rank Distribution and Percentage of Players- September. 2022 Esports Tales: Esports Tales; 2023 [30/05/2023]. Available from: https://www.esportstales.com/rainbow-six-siege/seasonal-rank-distribution-and-percentage-of-players.

[44] Milella V. Rocket League Rank Distribution and Percentage of Players- Season 9 Esports Tales: Esports Tales; 2023 [30/05/2023]. Available from: https://rocketleague.tracker.network/rocket-league/distribution?playlist=13.

[45] Milella V, PUBG Seasonal Rank Distribution and Percentage of Players Esports Tales. Esports Tales 2023 [30/05/2023]. Available from: https://www.esportstales.com/pubg/seasonal-rank-distribution-and-players-percentage-by-tier.

[46] Milella V, Teamfight Tactics (TFT). : Seasonal Rank System and Player Distribution- April 2023 Esports Tales: Esports Tales; 2023 [30/05/2023]. Available from: https://www.esportstales.com/teamfight-tactics/seasonal-rank-system-and-player-distribution.

[47] Hedlund D, Fried G, Smith R. Esports Business Management: Human Kinetics; 2020.

[48] IPAQ Research Committee. Guidelines for data processing and analysis of the International Physical Activity Questionnaire (IPAQ)-short and long forms. 2005. Available from: http://www.ipaq.ki.se/scoring.pdf.

[49] Craig CL, Marshall AL, Sjöström M, Bauman AE, Booth ML, Ainsworth BE (2003). International physical activity questionnaire: 12-country reliability and validity. Med Sci Sports Exerc.

[50] Markland D, Tobin V (2004). A modification to the behavioural regulation in exercise questionnaire to include an assessment of amotivation. J Sport Exerc Psychol.

[51] Wilson PM, Rodgers WM, Loitz CC, Scime G (2006). It’s who I Am… really!’The importance of integrated regulation in exercise contexts 1. J Appl Biobehavioral Res.

[52] Fairclough SJ, Noonan R, Rowlands AV, Van Hees V, Knowles ZOE, Boddy LM. Wear Compliance and Activity in Children Wearing Wrist- and Hip-Mounted Accelerometers. Medicine & Science in Sports & Exercise. 2016;48(2). Available from: https://journals.lww.com/acsm-msse/fulltext/2016/02000/wear_compliance_and_activity_in_children_wearing.9.aspx. 10.1249/MSS.0000000000000771 26375253

[53] MÂSse LC, Fuemmeler BF, Anderson CB, Matthews CE, Trost SG, Catellier DJ et al. Accelerometer Data Reduction: A Comparison of Four Reduction Algorithms on Select Outcome Variables. Medicine & Science in Sports & Exercise. 2005;37(11). Available from: https://journals.lww.com/acsm-msse/Fulltext/2005/11001/Accelerometer_Data_Reduction__A_Comparison_of_Four.7.aspx. 10.1249/01.mss.0000185674.09066.8a 16294117

[54] Crotti M, Foweather L, Rudd JR, Hurter L, Schwarz S, Boddy LM. Development of raw acceleration cut-points for wrist and hip accelerometers to assess sedentary behaviour and physical activity in 5–7-year-old children. Journal of Sports Sciences. 2020;38(9):1036-45. 10.1080/02640414.2020.1740469. 10.1080/02640414.2020.1740469 32228156

[55] Rhudy MB, Dreisbach SB, Moran MD, Ruggiero MJ, Veerabhadrappa P. Cut points of the Actigraph GT9X for moderate and vigorous intensity physical activity at four different wear locations. Journal of Sports Sciences. 2020;38(5):503 – 10. 10.1080/02640414.2019.1707956. 10.1080/02640414.2019.1707956 31865845

[56] Migueles JH, Rowlands AV, Huber F, Sabia S, van Hees VT. GGIR: A Research Community–Driven Open Source R Package for Generating Physical Activity and Sleep Outcomes From Multi-Day Raw Accelerometer Data. Journal for the Measurement of Physical Behaviour. 2019;2(3):188 – 96. Available from: https://journals.humankinetics.com/view/journals/jmpb/2/3/article-p188.xml; 10.1123/jmpb.2018-0063.

[57] Hildebrand M, Van Hees VT, Hansen BH, Ekelund ULF. Age Group Comparability of Raw Accelerometer Output from Wrist- and Hip-Worn Monitors. Medicine & Science in Sports & Exercise. 2014;46(9). Available from: https://journals.lww.com/acsm-msse/Fulltext/2014/09000/Age_Group_Comparability_of_Raw_Accelerometer.17.aspx. 10.1249/MSS.0000000000000289 24887173

[58] Sabia S, Cogranne P, van Hees VT, Bell JA, Elbaz A, Kivimaki M et al. Physical Activity and Adiposity Markers at Older Ages: Accelerometer Vs Questionnaire Data. Journal of the American Medical Directors Association. 2015;16(5):438.e7-.e13. Available from: https://www.sciencedirect.com/science/article/pii/S1525861015000936; 10.1016/j.jamda.2015.01.086. 10.1016/j.jamda.2015.01.086 PMC4417049 25752539

[59] Bell JA, Hamer M, van Hees VT, Singh-Manoux A, Kivimäki M, Sabia S. Healthy obesity and objective physical activity1–3. The American Journal of Clinical Nutrition. 2015;102(2):268 – 75. 10.3945/ajcn.115.110924. 10.3945/ajcn.115.110924 PMC4515867 26156738

[60] Menai M, Van Hees VT, Elbaz A, Kivimaki M, Singh-Manoux A, Sabia S (2017). Accelerometer assessed moderate-to-vigorous physical activity and successful ageing: results from the Whitehall II study. Sci Rep.

[61] Mukaka MM (2012). Statistics corner: a guide to appropriate use of correlation coefficient in medical research. Malawi Med J.

[62] Donaldson SI, Grant-Vallone EJ (2002). Understanding self-report bias in organizational behavior research. J Bus Psychol.

[63] Canning KL, Brown RE, Jamnik VK, Salmon A, Ardern CI, Kuk JL (2014). Individuals underestimate moderate and vigorous intensity physical activity. PLoS ONE.

[64] Guthold R, Stevens GA, Riley LM, Bull FC (2018). Worldwide trends in insufficient physical activity from 2001 to 2016: a pooled analysis of 358 population-based surveys with 1· 9 million participants. Lancet Global Health.

[65] Hallal PC, Gomez LF, Parra DC, Lobelo F, Mosquera J, Florindo AA (2010). Lessons learned after 10 years of IPAQ use in Brazil and Colombia. J Phys Activity Health.

[66] Ekelund U, Sepp H, Brage S, Becker W, Jakes R, Hennings M (2006). Criterion-related validity of the last 7-day, short form of the International Physical Activity Questionnaire in Swedish adults. Public Health Nutr.

[67] Ekelund U, Steene-Johannessen J, Brown WJ, Fagerland MW, Owen N, Powell KE et al. Does physical activity attenuate, or even eliminate, the detrimental association of sitting time with mortality? A harmonised meta-analysis of data from more than 1 million men and women. The Lancet. 2016;388(10051):1302-10. Available from: https://www.sciencedirect.com/science/article/pii/S0140673616303701; 10.1016/S0140-6736(16)30370-1. 10.1016/S0140-6736(16)30370-1 27475271

[68] Peterson NE, Sirard JR, Kulbok PA, DeBoer MD, Erickson JM (2018). Sedentary behavior and physical activity of young adult university students. Res Nurs Health.

[69] Roth G. Global Burden of Disease Collaborative Network. Global Burden of Disease Study 2017 (GBD 2017) Results. Seattle, United States: Institute for Health Metrics and Evaluation (IHME), 2018. The Lancet. 2018;392:1736-88.

[70] World Health Organisation. Noncommunicable diseases World Health Organisation: World Health Organisation. 2022 [03/11/2022]. Available from: https://www.who.int/news-room/fact-sheets/detail/noncommunicable-diseases.

[71] Katzmarzyk PT, Friedenreich C, Shiroma EJ, Lee IM. Physical inactivity and non-communicable disease burden in low-income, middle-income and high-income countries. British Journal of Sports Medicine. 2022;56(2):101. Available from: http://bjsm.bmj.com/content/56/2/101.abstract; 10.1136/bjsports-2020-103640. 10.1136/bjsports-2020-103640 PMC8478970 33782046

[72] Ekelund U, Tarp J, Steene-Johannessen J, Hansen BH, Jefferis B, Fagerland MW, et al. Dose-response associations between accelerometry measured physical activity and sedentary time and all cause mortality: systematic review and harmonised meta-analysis. BMJ. 2019;366. 10.1136/bmj.l4570. 10.1136/bmj.l4570 PMC6699591 31434697

[73] Paramitha ST, Hasan MF, Ilsya MNF, Anggraeni L, Ramadhan MG (2021). Level of physical activity of Indonesian esport athletes in the piala presiden esport 2019. Jurnal SPORTIF: Jurnal Penelitian Pembelajaran.

[74] Garriguet D, Tremblay S, Colley RC. Comparison of Physical Activity Adult Questionnaire results with accelerometer data. Health reports. 2015;26(7):11. Available from: https://pubmed.ncbi.nlm.nih.gov/26177042/. 26177042

[75] Downs A, Van Hoomissen J, Lafrenz A, Julka DL (2014). Accelerometer-measured versus self-reported physical activity in college students: implications for research and practice. J Am Coll Health.

[76] Nelson MC, Taylor K, Vella CA (2019). Comparison of self-reported and objectively measured sedentary behavior and physical activity in undergraduate students. Meas Phys Educ Exerc Sci.

[77] Rzewnicki R, Auweele YV, De Bourdeaudhuij I (2003). Addressing overreporting on the International Physical Activity Questionnaire (IPAQ) telephone survey with a population sample. Public Health Nutr.

[78] Ainsworth BE, Macera CA, Jones DA, Reis JP, Addy CL, Bowles HR (2006). Comparison of the 2001 BRFSS and the IPAQ Physical Activity questionnaires. Med Sci Sports Exerc.

[79] Sánchez-Herrera S, Cubero J, Feu S, Durán-Vinagre MÁ (2022). Motivation regarding Physical Exercise among Health Science University students. Int J Environ Res Public Health.

[80] Mahony R, Blake C, Matthews J, Donnoghue GO, Cunningham C (2019). Physical activity levels and self-determined motivation among future healthcare professionals: utility of the behavioral regulation in Exercise Questionnaire (BREQ-2). Physiother Theory Pract.

[81] Silva MN, Vieira PN, Coutinho SR, Minderico CS, Matos MG, Sardinha LB et al. Using self-determination theory to promote physical activity and weight control: a randomized controlled trial in women. Journal of Behavioral Medicine. 2010;33(2):110 – 22. 10.1007/s10865-009-9239-y. 10.1007/s10865-009-9239-y 20012179

[82] Burgess E, Hassmén P, Welvaert M, Pumpa K (2017). Behavioural treatment strategies improve adherence to lifestyle intervention programmes in adults with obesity: a systematic review and meta-analysis. Clin Obes.

[83] Manninen M, Dishman R, Hwang Y, Magrum E, Deng Y, Yli-Piipari S. Self-determination theory based instructional interventions and motivational regulations in organized physical activity: A systematic review and multivariate meta-analysis. Psychology of Sport and Exercise. 2022;62:102248. Available from: https://www.sciencedirect.com/science/article/pii/S1469029222001169; 10.1016/j.psychsport.2022.102248.

[84] Ahmadi A, Noetel M, Parker P, Ryan RM, Ntoumanis N, Reeve J (2023). A classification system for teachers’ motivational behaviors recommended in self-determination theory interventions. J Educ Psychol.

[85] Rogstad ET (2022). Gender in eSports research: a literature review. Eur J Sport Soc.

[86] Dunn R, Turkay S, Minett G, Johnson D, McNulty C. Press Start on Equity in Esports Research: identifying immediate research needs for female e’athletes: a scoping review. Women in Sport Congress; Sydney, Australia2024.

[87] Martins J, Marques A, Sarmento H, Carreiro da Costa F. Adolescents’ perspectives on the barriers and facilitators of physical activity: a systematic review of qualitative studies. Health Education Research. 2015;30(5):742 – 55. 10.1093/her/cyv042. 10.1093/her/cyv042 26324394

[88] Peng B, Ng JYY, Ha AS. Barriers and facilitators to physical activity for young adult women: a systematic review and thematic synthesis of qualitative literature. International Journal of Behavioral Nutrition and Physical Activity. 2023;20(1):23. 10.1186/s12966-023-01411-7. 10.1186/s12966-023-01411-7 PMC9972741 36849995

