# Supplementary material for: Physical Activity and Self-Determination towards Exercise among Esports Athletes
Source: Sports Med Open. 2024 Apr 16;10:40. doi: 10.1186/s40798-024-00700-0 (PMC11021385; doi:10.1186/s40798-024-00700-0)
Supplement: Supplementary file 1 — Supplementary Material 1 [file 40798_2024_700_MOESM1_ESM.pdf]

Physical Activity and Self-Determination towards Exercise among E'Athletes

Mitchell Nicholson[1], Courtney Thompson[1,2], Dylan Poulus[3], Toby Pavey[1], Rob Robergs[1], Vincent Kelly[1], Craig McNulty[1]

[1]School of Exercise and Nutrition Sciences, Faculty of Health, Queensland University of Technology, Brisbane, QLD, Australia

[2]School of Health, Faculty of Health, University of the Sunshine Coast, Sippy Downs, QLD, Australia

[3]School of Human Sciences, Faculty of Health, Southern Cross University, Coolangatta, QLD, Australia

Corresponding author: Mitchell Nicholson, mitchell.nicholson@hdr.qut.edu.au, Queensland University of Technology (QUT), Faculty of Health, School of Exercise and Nutrition Sciences, Victoria Park Road, Kelvin Grove, Queensland 4059, Australia.

Additional File 1: The Survey

Physical Activity and Self-Determination towards Exercise among E'Athletes
PARTICIPANT INFORMATION SHEET
QUT Ethics Approval Number: 1045

Research Team:
Principal Researcher:
Mitchell Nicholson, Ph.D. Candidate

Associate Researchers:
Dr. Craig McNulty, Principal Supervisor
Dr. Courtney Thompson
Assoc. Prof. Vincent G Kelly, Associate Supervisor
Assoc. Prof. Robert Robergs, Associate Supervisor
Assoc. Prof. Toby Pavey

School of Exercise and Nutrition Sciences, Faculty of Health, Queensland University of Technology (QUT)

Why is the study being conducted?
This research project is being undertaken as part of a Doctor of Philosophy study by Mitchell Nicholson.

The purpose of this research project is to gain a better understanding of the fitness attitudes that influence the physical activity of current esports players across a wide range of esports game titles. Currently, the physical activity behaviors of esports players are poorly understood. This research may identify certain areas that need to be addressed to help mitigate physical inactivity, poor health attitudes, and excessive sedentary times to ultimately reduce the risk of morbidity and mortality associated with these behaviors. This research will inform the direction of future research aiming to maximise player performance through the delivery of informed training.

You are invited to participate in this research project because you are a current amateur or professional esports player that is aged over 18 years of age.

What does participation involve?
Participation will involve completing a questionnaire that contains up to fifty-two to seventy-three questions with some questions using Likert scale answers (strongly agree – strongly disagree) that will take approximately 10-15 minutes of your time.

Your participation in this survey will be completely anonymous. At no stage are you required to provide your name and no data will be collecting regarding IP address. However, some questions about your

general demographic information will be asked (age, location, esports team). The survey will follow the following structure:

Demographic details (6 questions)
Esports Experience (7 questions)
International Physical Activity Questionnaires (14-29 questions)
The Behavioral regulation in Exercise Questionnaire 3 (BREQ-3; 24 questions)

Your participation in this research project is entirely voluntary. Any publication of this research will not present individual responses. If you agree to participate you do not have to complete any question(s) you are uncomfortable answering. If you do agree to participate you can withdraw from the research project during your participation without comment or penalty. Any information already obtained that can be linked to you will be destroyed. However, as the survey is anonymous once it has been submitted it will not be possible to withdraw your response.

What are the possible benefits for me if I take part?

It is expected that this research project will inform key stakeholders on the current attitudes that affect physical activity levels across a wide range of esports game titles. The outcomes of the research, however, may benefit the esports industry through informed training strategies to maximize competitive performance and health outcomes of esports players.

You will be provided with a summary of the findings once the project is complete.

What are the possible risks for me if I take part? There are no risks beyond normal day-to-day living associated with your participation in this research project. However, if you feel uncomfortable at any stage you can skip a question or withdraw from the survey. If you experience any discomfort during the survey, you can seek assistance or advice with the following community services which can provide professional assistance in times of distress:

Mental Health Resource Hub: https://www.wearespur.com/org/mentalhealthhub

This page contains a wide range of mental health services around the world.

What about privacy and confidentiality? All responses are anonymous i.e., it will not be possible to identify you at any stage of the research, because personal identifying information is not sought in any of the responses and no traceable information is collected via the server or survey tool.

Any data collected as part of this research project will be stored securely as per QUT's Management of research data policy. Data will be stored for a minimum of 5 years and can be disclosed if it is to protect you or others from harm, if specifically required by law, or if a regulatory or monitoring body such as the ethics committee requests it. Please note that non-identifiable data from this research project may be used as comparative data in future projects or stored on an open-access database for secondary analysis.

How do I give my consent to participate? The submission or return of the completed survey is accepted as an indication of your consent to participate in this research project.

What if I have questions about the research project? If you have any questions or require further information, please contact one of the listed researchers:

What if I have a concern or complaint regarding the conduct of the research project? QUT is committed to research integrity and the ethical conduct of research projects. If you wish to discuss the study with someone not directly involved, particularly in relation to matters concerning policies, information, or complaints about the conduct of the study or your rights as a participant, you may contact the QUT Research Ethics Advisory Team [redacted] or email.

Research is currently being undertaken by Exercise Scientists and Exercise Physiologists within competitive esports at QUT in Australia. If you live in Australia and would like updates on future esports research projects at QUT please email [redacted] for more information!

Page Break

Q2 Are you older than 18-years of age?

- ◯ No
- ◯ Yes

Q3 How old are you in years?

____

Q4 What gender do you identify as?

- ◯ Male
- ◯ Female
- ◯ Non-binary / third gender
- ◯ Prefer to describe myself as ____
- ◯ Prefer not to say

Q5 What is your current height in centimeters? (cm)

____

Q6 What is your current weight in kilograms? (kg)

____

Q7 What best describes your current occupation status?

- Employed full time
- Employed part time
- Unemployed looking for full-time work
- Unemployed looking for part-time work
- Unemployed not looking for work
- Retired
- Student
- Unemployed
- Prefer not to say

Q8 What is the highest level of education you have completed?

- ◯ No schooling
- ◯ Pre School
- ◯ Primary Education
- ◯ Secondary Education
- ◯ Tertiary Education
- ◯ University or other higher education
- ◯ Other type of education

End of Block: Demographics

Start of Block: Country

Q9 In which country do you currently reside?

▼ Afghanistan ... Zimbabwe

End of Block: Country

Start of Block: Esports Information

Q10 What Esports title are you currently highest ranked in?

◯ League of Legend (LoL)

◯ Counter Strike: Global Offensive (CS:GO)

◯ Overwatch

◯ Valorant

◯ Apex Legends

◯ DOTA 2

◯ Rainbow Six Siege

◯ StarCraft II

◯ Hearthstone

◯ Rocket League

◯ Player Unknown Battle Grounds (PUBG)

◯ Teamfight Tactics

◯ Other (please enter below) ____

Q11 What is your in-game League of Legends Rank?

▼ Iron 4 ... Challenger

Q12 What is your in-game Counter Strike: Global Offensive Rank?

▼ Silver 1 ... The Global Elite

Q13 What is your in-game Overwatch Rank?

▼ Bronze ... Grandmaster

Q14 What is your current Skill Rating (SR)?

____

Q15 What is your in-game Valorant Rank?

▼ Iron 1 ... Radiant

Q16 What is your in-game Apex Legends Rank?

▼ Bronze 4 ... Apex Predator

Q17 What is your current Apex Legends Ranked Points (RP)?

____

Q18 What is your in-game Rainbow Six Siege Rank?

▼ Copper 5 ... Champion

Q19 What is your in-game DOTA 2 Rank?

▼ Herald 1 ... Immortal

Q20 What is your in-game StarCraft II Rank?

▼ Bronze 3 ... Master 1

Q21 What is your in-game Hearthstone Rank?

▼ Bronze 10 ... Legend

Q22 What type of Rocket-League do you play?

- ○ Solo Duel
- ○ Standard
- ○ Doubles

Q23 What is your current rank in that Rocket-League playlist?

▼ Bronze 1 ... Supersonic Legend

Q24 What game mode are you ranked highest in for Player Unknown Battle Grounds?

◯ Solo

◯ Squad

Q25 What is your in-game Player Unknown Battle Grounds rank?

▼ Bronze ... Grandmaster

Q26 What is your in-game Teamfight Tactics rank?

▼ Iron 4 ... Challenger

Page Break

Q27 Which level of Esport best describes your current level of competition?

○ Casual Esports (e.g., playing esports recreationally or sometimes competitively)

○ Youth Esports (e.g., new to esports playing for pure enjoyment and sometimes competitively)

○ High School Esports (e.g., participating/competing for a high school team or program)

○ Collegiate/Intervarsity Esports (e.g., participating/competing for a collegiate/intervarsity team, which may include a scholarship or prize money)

○ Professional Esports (e.g., participating/competing within a gaming organisation/team for prize money or a salary)

Q28 Do you play for a gaming company/organisation?

○ No

○ Yes (enter team/ organisation name) ____

○ Prefer not to say

Q29 What is your highest cash prize won whilst competing? (USD)

○ Amount in USD ____

○ None

○ Prefer not to say

Page Break

End of Block: Esports Information

Start of Block: International Physical Activity Questionnaire (IPAQ)

Q30 **International Physical Activity Questionnaire (IPAQ)** We are interested in finding out about the kinds of physical activities that people do as part of their everyday lives. The questions will ask you about the time you spent being physically active in the **last 7 days.** Please answer each question even if you do not consider yourself to be an active person. Please think about the activities you do at work, as part of your house and yard work, to get from place to place, and in your spare time for recreation, exercise, or sport.

Think about all the **vigorous activities** that you did i**n the last 7 days.** Vigorous physical activities refer to activities that take hard physical effort and make you breathe much harder than normal.
Moderate activities refer to activities that take moderate physical effort and make you breathe somewhat harder than normal. Think only about those physical activities that you did for at least 10 minutes at a time.

Page Break

Q31 **PART 1: JOB-RELATED PHYSICAL ACTIVITY**

The first section is about your work. This includes paid jobs, farming, volunteer work, course work, and any other unpaid work that you did outside your home. Do not include unpaid work you might do around your home, like housework, yard work, general maintenance, and caring for your family. These are asked in Part 3.

Q32 Do you currently have a job or do any unpaid work outside your home?

- ○ Yes
- ○ No

Q33 During the last **7 days**, on how many days did you do **vigorous** physical activities like heavy lifting, digging, heavy construction, or climbing upstairs **as part of your work?**

Think about only those physical activities that you did for at least 10 minutes at a time.

Days per week:

- ○ 1 days
- ○ 2 Days
- ○ 3 Days
- ○ 4 Days
- ○ 5 Days
- ○ 6 Days
- ○ 7 Days
- ○ None

Q34 How much time did you usually spend on one of those days doing **vigorous** physical activities as part of your work?

	Hours	Minutes
Time		

Q35 Again, think about only those physical activities that you did for at least 10 minutes at a time. During the **last 7 days,** on how many days did you do **moderate** physical activities like carrying light loads **as part of your work?** Please do not include walking.

Days per week:

- 1 Days
- 2 Days
- 3 Days
- 4 Days
- 5 days
- 6 Days
- 7 Days
- None

Q36 How much time did you usually spend on one of those days doing **moderate** physical activities as part of your work?

	Hours	Minutes
Time		

Q37 During the **last 7 days**, on how many days did you **walk** for at least 10 minutes at a time **as part of your work?** Please do not count any walking you did to travel to or from work.

Days per week:

- 1 Days
- 2 Days
- 3 Days
- 4 Days
- 5 days
- 6 Days
- 7 Days
- None

Q38 How much time did you usually spend on one of those days walking as part of your work?

	Hours	Minutes
Time		

Page Break

Q39 **PART 2: TRANSPORTATION PHYSICAL ACTIVITY**

The following questions are about how you traveled from place to place, including to places like work, stores, movies, and so on.

Q40 During the **last 7 days,** on how many days did you **travel in a motor vehicle** like a train, bus, car, electric scooter, or tram?

Days per week:

- ○ 1 Days
- ○ 2 Days
- ○ 3 Days
- ○ 4 Days
- ○ 5 days
- ○ 6 Days
- ○ 7 Days
- ○ None

Q41 How much time did you usually spend on one of those days **traveling** in a train, bus, car, tram, or other kind of motor vehicle?

	Hours	Minutes
Time		

Q42 Now think only about the cycling and walking you might have done to travel to and from work, to do errands, or to go from place to place.

During the **last 7 days,** on how many days did you **bicycle** for at least 10 minutes at a time to go **from place to place?**

Days per week:

- ◯ 1 Days
- ◯ 2 Days
- ◯ 3 Days
- ◯ 4 Days
- ◯ 5 days
- ◯ 6 Days
- ◯ 7 Days
- ◯ None

Q43 How much time did you usually spend on one of those days to **bicycle** from place to place?

	Hours	Minutes
Time		

Q44 During the **last 7 days,** on how many days did you **walk** for at least 10 minutes at a time to go **from place to place?**

Days per week:

- 1 Days
- 2 Days
- 3 Days
- 4 Days
- 5 days
- 6 Days
- 7 Days
- None

Q45 How much time did you usually spend on one of those days to **walking** from place to place?

	Hours	Minutes
Time		

Page Break

Q46 **PART 3: HOUSEWORK, HOUSE MAINTENANCE, AND CARING FOR FAMILY**

This section is about some of the physical activities you might have done in the **last 7 days** in and around your home, like housework, gardening, yard work, general maintenance work, and caring for your family.

Q47 Think about only those physical activities that you did for at least 10 minutes at a time. During the **last 7 days,** on how many days did you do **vigorous physical activities** like heavy lifting, chopping wood, shoveling snow, or digging **in the garden or yard?**

Days per week:

- ○ 1 Days
- ○ 2 Days
- ○ 3 Days
- ○ 4 Days
- ○ 5 days
- ○ 6 Days
- ○ 7 Days
- ○ None

Q48 How much time did you usually spend on one of those days doing vigorous physical activities in the garden or yard?

	Hours	Minutes
Time		

Q49 Again, think about only those physical activities that you did for at least 10 minutes at a time. During the **last 7 days**, on how many days did you do **moderate** activities like carrying light loads, sweeping, washing windows, and raking **in the garden or yard?**

Days per week:

- 1 Days
- 2 Days
- 3 Days
- 4 Days
- 5 days
- 6 Days
- 7 Days
- None

Q50 How much time did you usually spend on one of those days doing **moderate** physical activities in the garden or yard?

	Hours	Minutes
Time		

Q51 Again, think about only those physical activities that you did for at least 10 minutes at a time. During the **last 7 days**, on how many days did you do **moderate** activities like carrying light loads, sweeping, washing windows, and raking **in the garden or yard?**

Days per week:

- ○ 1 Days
- ○ 2 Days
- ○ 3 Days
- ○ 4 Days
- ○ 5 days
- ○ 6 Days
- ○ 7 Days
- ○ None

Q52 How much time did you usually spend on one of those days doing **moderate** physical activities inside your home?

	Hours	Minutes
Time		

Page Break

Q53 **PART 4: RECREATION, SPORT, AND LEISURE-TIME PHYSICAL ACTIVITY**

This section is about all the physical activities that you did in the **last 7 days** solely for recreation, sport, exercise or leisure. Please do not include any activities you have already mentioned.

Q54 Not counting any walking you have already mentioned, during the **last 7 days,** on how many days did you **walk** for at least 10 minutes at a time **in your leisure time?**

Days per week:

- ○ 1 Days
- ○ 2 Days
- ○ 3 Days
- ○ 4 Days
- ○ 5 days
- ○ 6 Days
- ○ 7 Days
- ○ None

Q55 How much time did you usually spend on one of those days **walking** in your leisure time?

	Hours	Minutes
Time		

Q56 Think about only those physical activities that you did for at least 10 minutes at a time. During the **last 7 days,** on how many days did you do **vigorous** physical activities like aerobics, running, fast bicycling, or fast swimming **in your leisure time?**

Days per week:

- ◯ 1 Days
- ◯ 2 Days
- ◯ 3 Days
- ◯ 4 Days
- ◯ 5 days
- ◯ 6 Days
- ◯ 7 Days
- ◯ None

Q57 How much time did you usually spend on one of those days doing **vigorous** physical activities in your leisure time?

	Hours	Minutes
Time		

Q58 Again, think about only those physical activities that you did for at least 10 minutes at a time. During the **last 7 days**, on how many days did you do **moderate** physical activities like bicycling at a regular pace, swimming at a regular pace, and doubles tennis **in your leisure time?**

Days per week:

- ◯ 1 Days
- ◯ 2 Days
- ◯ 3 Days
- ◯ 4 Days
- ◯ 5 days
- ◯ 6 Days
- ◯ 7 Days
- ◯ None

Q59 How much time did you usually spend on one of those days doing **moderate** physical activities in your leisure time?

	Hours	Minutes
Time		

Page Break

Q60 **PART 5: TIME SPENT SITTING**

The last questions are about the time you spend sitting while at work, at home, while doing course work and during leisure time. This may include time spent sitting at a desk, visiting friends, reading or sitting or lying down to watch television. The questions will also ask you about how much time you spend playing video games, this can either be practicing competitively or playing leisurely. Do not include any time spent sitting in a motor vehicle that you have already told me about.

Q61 During the last **7 days,** how much time did you usually spend **sitting playing video games** on a **weekday?**

	Hours	Minutes
Time		

Q62 During the last **7 days,** how much time did you usually spend **sitting playing video games** on a **weekend?**

	Hours	Minutes
Time		

Q63 During the last **7 days,** how much time did you usually spend **sitting** on a **weekday?** (Other activities: watching TV, course work, reading etc.)

	Hours	Minutes
Time		

Q64 During the last **7 days,** how much time did you usually spend **sitting** on a **weekend?** (Other activities: watching TV, course work, reading etc.)

	Hours	Minutes
Time		

End of Block: International Physical Activity Questionnaire (IPAQ)

Start of Block: BREQ-3

Q65 **Why do you engage in exercise?**

We are interested in the reasons underlying peoples' decisions to engage or not engage in physical exercise. Using the scale below, please indicate to what extent each of the following items is true for you. Please note that there are no right or wrong answers and no trick questions. We simply want to know how you personally feel about exercise. Your responses will be held in confidence and only used for our research purposes.

Q66 Using the scale below, please indicate to what extent each of the following items is true for you.

	Not True for me	-	Sometimes true for me	-	Very True for me
It’s important to me to exercise regularly	○	○	○	○	○
I don’t see why I should have to exercise	○	○	○	○	○
I exercise because it’s fun	○	○	○	○	○
I feel guilty when I don’t exercise	○	○	○	○	○
I exercise because it is consistent with my life goals	○	○	○	○	○
I exercise because other people say I should	○	○	○	○	○
I value the benefits of exercise	○	○	○	○	○
I can’t see why I should bother exercising	○	○	○	○	○
I enjoy my exercise sessions	○	○	○	○	○
I feel ashamed when I miss an exercise session	○	○	○	○	○
I consider exercise part of my identity	○	○	○	○	○
I take part in exercise because my friends/family/partner say I should	○	○	○	○	○
I think it is important to make the effort to exercise regularly	○	○	○	○	○
I don’t see the point in exercising	○	○	○	○	○

I find exercise a pleasurable activity	○	○	○	○	○
I feel like a failure when I haven’t exercised in a while	○	○	○	○	○
I consider exercising a fundamental part of who I am	○	○	○	○	○
I exercise because others will not be pleased with me if I don't	○	○	○	○	○
I get restless if I don’t exercise regularly	○	○	○	○	○
I think exercising is a waste of time	○	○	○	○	○
I get pleasure and satisfaction from participating in exercise	○	○	○	○	○
I would feel bad about myself if I was not making time to exercise	○	○	○	○	○
I consider exercise consistent with my values	○	○	○	○	○
I feel under pressure from my friends/family to exercise	○	○	○	○	○

End of Block: BREQ-3

Additional File 1: Table 1: Frequency table of player country of residence for survey participants

	**Countries with 1 Participant (*n*)**	**Countries with between 2 and 10 Participants (*n*)**	**Countries with between 11 and 25 Participants (*n*)**	**Countries with more than 25 Participants (*n*)**
Country name	Albania (1), Bahrain (1), Congo (1), Costa Rica (1), Cyprus (1), Hong Kong (1), Latvia (1), Moldova (1), Mongolia (1), Montenegro (1), Morocco (1), Pakistan (1), Portugal (1), Thailand (1), Turkey (1), Ukraine (1), United Arab Emirates (1), Vietnam (1)	Brazil (10), India (9), Belgium (8), Czech Republic (8), Norway (8), Philippines (8), Russia (8), Malaysia (7), Denmark (6), Ireland (5), Switzerland (5), Hungary (5), Italy (4), Mexico (4), Singapore (3), Spain (4), Austria (3), Bulgaria (3), Croatia (3), Greece (3), Iceland (3), Indonesia (3), Lithuania (3), Romania (3), South Africa (3), South Korea (3), Afghanistan (2), Andorra (2), Argentina (2), China (2), Japan (2), Serbia (2), Slovakia (2), Taiwan (2)	Sweden (18), France (18), Netherlands (17), Finland (12), Poland (12)	United States (244), Australia (113), Germany (53), Canada (47), United Kingdom (44)
Participants (*n*)	18	146	77	501
Number of Countries	19	35	5	5
